# The impact of preoperative breast biopsy on the risk of sentinel lymph node metastases: analysis of 2502 cases from the Austrian sentinel node biopsy study group

**DOI:** 10.1038/sj.bjc.6602205

**Published:** 2004-10-12

**Authors:** C Peters-Engl, P Konstantiniuk, C Tausch, A Haid, B Hoffmann, M Jagoutz-Herzlinger, F Kugler, S Redtenbacher, S Roka, P Schrenk, D Steinmassl

**Affiliations:** 1 Department of Gynaecology and Obstetrics, Krankenhaus Lainz, Wolkersbergenstrasse 1, A-1130 Vienna, Austria; 2 Department of Vascular Surgery, University of Graz, Austria; 3 Department of Surgery, Barmherzige Schwestern Hospital, Linz, Austria; 4 Department of Surgery, Landeskrankenhaus Feldkirch, Austria; 5 2nd Department of Surgery, Wilhelminenspital, Vienna, Austria; 6 Department of Pathology, Krankenhaus Lainz, Vienna, Austria; 7 Department of General Surgery, University of Vienna, Austria; 8 2nd Department of Surgery, Allgemeines Krankenhaus Linz, Austria; 9 Department of Surgery, Krankenhaus Kufstein, Austria

**Keywords:** preoperative breast biopsy, sentinel node, metastases

## Abstract

Preoperative breast biopsy might cause disaggregation of tumour cells and tumour cell spread. The purpose of this study was to investigate the impact of preoperative biopsy on the rate of metastases to the sentinel lymph node (SLN) of patients with primary breast cancer. We report the results of 2502 patients with primary breast cancer, who were operated, and a sentinel node biopsy was performed. The association of preoperative biopsy with the risk of SLN metastases was examined by regression analyses and tested for possible confounding well-known factors for axillary node metastases. In all, 1890 patients were available for final analyses; 1048 (55.4%) patients had a preoperative diagnosis performed by fine-needle aspiration or core biopsy; 641 (33.9%) patients had a positive SLN when conventional H&E and IHC staining was performed. Patients with preoperative breast biopsy showed a 1.37 times (95% CI, 1.13–1.66) increased risk of SLN metastases on univariate analysis, but this result was not persistent when analysis was adjusted for other relevant factors for axillary node metastases, OR 1.09 (95% CI, 0.85–1.40). In addition, subgroup analyses of the risk for occult micro metastases to the SLN (detected by IHC only) on H&E-negative cases also showed no increased risk associated with preoperative biopsy, OR 1.07 (95% CI, 0.69–1.65). The conclusion, based on the present data, is that preoperative breast biopsy does not cause artificial tumour cell spread to the SLN, with possible negative impact on the prognosis of breast cancer.

Modern surgical treatment for breast tumours requires a preoperative diagnosis of malignancy (Perry, 2001). Fine-needle aspiration and core biopsy are widely used for evaluation of palpable and nonpalpable suspicious breast lesions. However, there has been serious concern about malignant tumour cell displacement promoting iatrogenic tumour spread. Tumour cell displacement rates to the needle tract of up to 30% have been reported (Youngson *et al*, 1995; Diaz *et al*, 1999). In theory, tumour seeding into lymphatic or vascular vessels would carry the same risk of axillary lymph node metastases as true lymphatic invasion. To our knowledge, no study has investigated the rate of breast cancer cell seeding to the axillary nodes for fine-eedle aspiration and large gauge needle biopsy procedures. The concept of sentinel lymphadenectomy has been demonstrated to be an accurate staging alternative for breast cancer (Krag *et al*, 1993, 1998; Giuliano *et al*, 1994, 1997; Veronesi *et al*, 1997; Veronesi *et al*, 2003). The sentinel node (SLN) is as per definition ‘the first lymph node that receives afferent lymphatic drainage from a primary tumour’. With the thorough pathologic examination of the SLN, it is possible to detect even early tumour cell spread in a lymph node, which might not have been seen otherwise. The previous analysis carried out by the Austrian Sentinel Node Biopsy Study Group revealed a nonsignificant trend of an increased risk of SLN metastasis after preoperative breast biopsy (Pichler-Gebhard *et al*, 2002). The purpose of this study was to evaluate the impact of preoperative biopsy on the rate of metastasis to the SLN of patients with primary breast cancer.

## MATERIALS AND METHODS

### Patients and data collection

A total of 2502 consecutive women with primary breast cancer, in whom a SLN procedure was performed, were registered by the multi-centre database project (MCDBP) (Konstantiniuk *et al*, 2001). Patient data from 12 participating departments of the Austrian Sentinel Node Biopsy Study Group were collected prospectively from 1999 onwards, but data from some centres were obtained retrospectively from 1996 onwards. Feasibility and validation of the SLN biopsy method of the participating centres have been demonstrated by the Austrian Sentinel Node Biopsy Study Group previously. Each participating centre had to undergo a learning period, as has been established by means of quality control (Pichler-Gebhard *et al*, 2002). In all, 2328 cases remained after excluding women having received preoperative systemic treatment, patients with multifocal disease and *in situ* carcinomas as the role of SLN biopsy method in this group of patients still needs to be defined.

### Treatment methods

Biopsy procedures of palpable masses were carried out by fine-needle aspiration or automated gun, nonpalpable masses, either by sonographically or by stereotactically guided biopsy. The number of FNA or core specimens obtained was at the discretion of the examiner performing the procedure. In cases of nonpalpable lesions, a stereotactic or ultrasound-guided wire localisation of the tumour was performed preoperatively. Lymphatic mapping and SLN dissection (SLND) were performed by using blue dye or radiolabelled colloid, or a combination of both. Blue dye and colloid were injected either around the edge of the lesion or submammillarily. If an excisional biopsy had to be performed for the diagnosis of malignancy, confirmation by the use of frozen section was followed by immediate SLND in the majority of cases. Sentinel lymph node dissection was performed as a second procedure if a permanent section of the excision specimen revealed invasive tumour in frozen section-negative cases or when frozen section was not available. In patients with preoperative diagnosis of malignancy by core needle biopsy, SLND was performed upfront. Sentinel nodes were dissected and sent for frozen section. Standard surgical treatment with quadrantectomy or total mastectomy was completed. Axillary lymph node dissection was performed if frozen section of the SLN identified tumour cells. Tumours were classified as described by the American Joint Committee on Cancer. Sentinel nodes were examined at two-step section levels of the paraffin block separated by 250 *μ*m. One of every pair was stained at each level by H&E and followed by cytokeratin IHC staining with monoclonal anticytokeratin antibodies if the H&E sections did not reveal metastases. Every second corresponding slide was further stained with IHC (Rudas *et al*, 2002).

### Statistical analyses

Continuous variables were compared by Student's *t*-test. For comparison of categorical variables, the *χ*^2^ test was used. Risk estimates were carried out by univariate and multivariate logistic regression analysis. All reported *P*-values are results of two-sided tests. A *P*-value equal to or less than 5% was considered statistically significant. The SPSS 10.0.7 statistical software system was used for calculations.

## RESULTS

In 2079 out of 2328 patients a SLN was found, resulting in an overall identification rate of 89.3% (not stratified according to learning period). Finally, we had 1890 patients aged between 23 and 96 years (median age, 60 years) with complete information on all data evaluable for analyses. In all, 230 (12.1%) patients had a preoperative diagnosis carried out by FNA, 818 (43.3%) by core biopsy *vs* 842 (44.6%) patients without preoperative biopsy.

Patients' clinicopathological characteristics are listed in Table 1

**Table 1:** Patients characteristics

	**No biopsy**	**Biopsy**	
	***N*=842**	***N*=1048**	***P*-value**
Age (years, mean, s.d.)	60.4 (13.0)	59.7 (12.9)	0.242
Tumour size (mm, mean, s.d., range)	14.9 (s.d. 8.1, 1.0–75)	17.8 (s.d. 8.9, 0.5–80)	<0.0001
			
*Tumour location*			0.351
Inner upper	100 (11.9%)	140 (13.4%)	
Inner lower	54 (6.4%)	61 (5.8%)	
Outer upper	464 (55.1%)	604 (57.6%)	
Outer lower	135 (16.0%)	155 (14.8%)	
Central	89 (10.6%)	88 (8.4%)	
			
*Histologic type*			0.006
Ductal	526 (62.5%)	704 (67.2%)	
Lobular	97 (11.5%)	138 (13.2%)	
Ducto- lobular	90 (10.7%)	73 (7.0%)	
Other	129 (15.3%)	133 (12.7%)	
			
*Grading*			0.055
GI	149 (17.7%)	144 (13.7%)	
GII	439 (52.1%)	562 (53.6%)	
GIII	254 (30.2)	342 (32.6)	
			
*Learning period*			<0.0001
Yes	237 (28.1%)	177 (16.9%)	
No	607 (71.9%)	871 (83.1%)	
			
*Palpable*			<0.0001
No	352 (41.8%)	268 (25.6%)	
Yes	490 (58.2%)	780 (74.4%)	
			
*Department*			<0.0001
1	75 (8.9%)	177 (16.9%)	
2	40 (4.8%)	72 (6.9%)	
3	229 (27.2%)	143 (13.6%)	
4	188 (22.3%)	95 (9.0%)	
5	137 (16.3%)	338 (32.2%)	
6	63 (7.5%)	1 (0.1%)	
7	13 (1.5%)	55 (5.2%)	
8	40 (4.8%)	103 (9.8%)	
9	14 (1.7%)	30 (2.8%)	
10	10 (1.2%)	19 (1.8%)	
11	0 (0.0%)	13 (1.2%)	
12	33 (3.9%)	2 (0.2%)	
			
*SLN technique*			<0.0001
Blue dye	317 (37.6%)	184 (17.6%)	
Radioisotope	243 (28.9%)	146 (13.9%)	
Combination	282 (33.5%)	718 (68.5%)	

. Patients undergoing a preoperative biopsy had larger (mean difference 2.87 mm) and more palpable tumours than the control group. This might be due to the nonavailability of stereotactic procedure in some participating centres. Lymphatic mapping using blue dye alone was more common in the nonbiopsy group. We found more ductal and lobular carcinomas and less ducto-lobular and other subtypes in the biopsy group. Tumour grading was nearly evenly distributed; patients' age and tumour location were comparable between both groups. Overall, 7.4% of the patients had SLND performed as a second procedure (3.1% in the biopsy group *vs* 12.8% in the nonbiopsy group).

Sentinel lymph node metastases were detected in 641 (33.9%) of 1890 patients. In all, 388 (37.0%) positive SLN were observed in the preoperative biopsy group and 253 (30.0%) in the control group. A total of 553 (29.3%) patients had a positive SLN when conventional H&E staining was performed. In 88 (4.6%) patients the presence of metastases was detected by IHC only (Table 2

**Table 2:** SLN positivity according to biopsy and histological workup

	**No biopsy**	**Biopsy**	**FNA**	**Core biopsy**
	***N*=842**	***N*=1048**	***N*=230**	***N*=818**
SLN positivity (HE+IHC)	253 (30.0%)	388 (37.0%)	78 (33.9%)	310 (37.9%)
SLN positivity (HE)	213 (25.3%)	340 (32.4%)	73 (31.7%)	267 (32.6%)
SLN positivity (IHC)	40 (4.7%)	48 (4.6%)	5 (2.2%)	43 (5.3%)

).

With regard to the presence of a positive sentinel node (H&E and IHC staining), univariate regression analysis identified larger tumour size (*P*<0.0001), age (*P*=0.002), preoperative biopsy (*P*=0.001), histological type (*P*<0.0001), grading (*P*<0.0001), palpability of the lesion (*P*<0.0001), timing of SLN biopsy (*P*=0.008) and participating centre (*P*=0.003) as significant factors. Preoperative biopsy revealed a hazard ratio of 1.37 (95% CI, 1.13–1.66). Finally, all factors were entered in a multivariate regression model. The impact of each of the evaluated factors is shown in Table 3

**Table 3:** Association between SLN metastases and clinicopathologic factors by univariate and multivariate analysis

			**Univariate**			**Multivariate**		
	***N***	**% SLN positive**	**Odds ratio**	**95% CI**	***P*-value**	**Odds ratio**	**95% CI**	***P*-value**
Age (years)			0.98	0.98–0.99	0.002	0.98	0.97–0.99	<0.0001
Tumour size (mm)			1.07	1.06–1.09	<0.0001	1.06	1.05–1.08	<0.0001
								
*Histology*					<0.0001			0.0004
Ductal	1230	33	1.00			1.00		
Ducto-lobular	163	49	1.97	1.42–2.74	<0.0001	2.16	1.48–3.16	0.0001
Lobular	235	39	1.29	0.97–1.72	0.081	1.12	0.82–1.54	0.487
Others	262	25	0.68	0.51–0.93	0.016	0.82	0.58–1.16	0.246
								
*Grading*					<0.0001			0.0003
I	293	18	0.34	0.24–0.48	<0.0001	0.55	0.32–0.81	0.002
II	1001	36	0.89	0.72–1.10	0.275	1.17	0.93–1.48	0.177
III	596	39	1.00			1.00		
								
*Location*					0.710			0.310
Inner upper	240	30	1.00			1.00		
Inner lower	115	29	0.96	0.59–1.56	0.863	1.09	0.65–1.84	0.738
Outer upper	1068	33	1.19	0.88–1.62	0.252	1.26	0.91–1.76	0.165
Outer lower	290	38	1.43	0.99–2.06	0.053	1.49	1.01–2.22	0.044
Central	177	40	1.59	1.06–2.40	0.025	1.39	0.89–2.17	0.151
								
*Palpability*								
Palpable	1270	41	2.78	2.22–3.49	<0.0001	1.77	1.37–2.29	<0.0001
Not palpable	620	20	1.00			1.00		
								
*Preop-biopsy*								
No	842	30	1.000			1.00		
Yes	1048	37	1.37	1.13–1.66	0.001	1.09	0.85–1.40	0.508
								
*Learning period*								
No	1476	34	0.95	0.76–1.20	0.673	1.13	0.79–1.62	0.509
Yes	414	35	1.00					
								
*Department*					0.003			0.001
1	252	37	1.00			1.00		
2	112	42	1.24	0.78–1.95	0.360	1.75	1.04–2.95	0.036
3	372	29	0.71	0.50–0.99	0.047	0.75	0.50–1.12	0.157
4	283	41	1.20	0.85–1.71	0.294	0.75	0.47–1.18	0.210
5	475	28	0.66	0.48–0.92	0.014	0.56	0.38–0.83	0.004
6	64	33	0.83	0.47–1.49	0.543	0.52	0.24–1.09	0.084
7	68	32	0.82	0.46–1.44	0.488	0.74	0.39–1.40	0.354
8	143	43	1.27	0.84–1.93	0.260	1.35	0.84–2.16	0.216
9	44	36	0.98	0.50–1.90	0.945	1.02	0.45–2.30	0.958
10	29	34	0.90	0.40–2.02	0.798	0.88	0.3–2.32	0.795
11	13	31	0.76	0.23–2.54	0.655	0.74	0.20–2.81	0.664
12	35	25	0.51	0.22–1.16	0.108	0.49	0.19–1.29	0.150
								
*SLN technique*					0.087			0.175
Combination	1000	33.5	1.00					
Blue dye	501	38	1.27	1.01–1.59	0.037	1.20	0.86–1.68	0.287
Radiotracer	389	32	0.99	0.77–1.28	0.969	1.35	0.96–1.90	0.083
								
*SLN timing*								
1st procedure	1750	35	1.00			1.00		
2nd procedure	140	23	0.59	0.39–0.87	0.008	1.01	0.63–1.62	0.969
								
*Study period*								
Prospective	1354	34	1.00			1.00		
Retrospective	536	35	1.06	0.86–1.31	0.574	0.97	0.70–1.38	0.848

. Of the variables which showed a significant correlation with SLN positivity in the univariate analysis, tumour size, tumour grading, histological type, age, palpability and participating centre remained as independent predictors for SLN metastasis, whereas preoperative biopsy failed to show significance. Patients with preoperative breast biopsy had a nonsignificant 1.09-fold (95% CI, 0.85–1.40) increased risk for SLN metastases.

As seeding to the needle tract during FNA is a very rare event, patients with preoperative FNA might have a different rate of tumour cell seeding than patients with large-gauge needle biopsy (Roussel and Dalion, 1989; Tabara *et al*, 1991; Bott *et al*, 1999). Therefore, further analysis was carried out with FNA and core biopsy as separate categories. Univariate analysis showed a significant correlation with SLN positivity for core biopsy, OR 1.42 (95% CI, 1.16–1.74), whereas no impact for FNA RR 1.19 (95% CI, 0.88–1.63) was found. However, when analysis was adjusted for other relevant factors for axillary node metastasis, preoperative biopsy again failed to show significance, OR 1.10 (95% CI, 0.85–1.43) and OR 1.03 (95% CI, 0.69–1.53), respectively. In addition, subgroup analysis of the risk for occult micro-metastases to the SLN (detected by IHC only) on H&E-negative cases with additional IHC staining also failed to show an increased risk for preoperative biopsy, OR 1.07 (95% CI, 0.69–1.65).

## DISCUSSION

This study examined the risk of SLN metastases in patients who underwent prior FNA or large-gauge needle core biopsy. Our results do not confirm the findings of a recently published study, performed on a much smaller number of patients (Hansen *et al*, 2004), suggesting a positive association between needle biopsy and SLN metastases. Studies of SLN metastases must take into account all well-known predictive factors for axillary node metastases such as tumour size, clinical and histo-pathological criteria. The significant result of an increased risk for SLN metastases after preoperative biopsy obtained on univariate calculation was no longer present when adjustment for relevant predictors for axillary node metastases was done by means of multivariate analysis. The additional use of IHC for further evaluation of the SLN increases the detection of occult micro-metastases and improves the sensitivity of the SLN procedure (Czerniecki *et al*, 1999). We found a 6.5% conversion rate of H&E-negative patients to lymph node positive in our series. Nevertheless, patients who underwent a preoperative biopsy had no increased risk for a SLN metastases detected by H&E and IHC or IHC alone. Large tumours were more likely to have nodal involvement than smaller tumours; this is in agreement with other findings (Gann *et al*, 1999). Clinical palpability remains to be highly predictive for SLN metastases, as has been demonstrated by other authors (Silverstein *et al*, 2001). In addition to our previous findings, we found high-grade lesions and age to be associated with higher rate of metastases, which is in accordance with other studies (Gann *et al*, 1999). Infiltrating ducto-lobular carcinomas had higher rates of SLN metastasis than other types; this confirmed the surprising findings of our first analysis.

Without any doubt, the rate of metastases to the sentinel node can be affected by several factors. In order to objectify this study as far as possible, we identified five possibilities of biases, which may have adversely affected the accuracy of our risk assessment.

Firstly, no data on failure of preoperative core biopsy were available in our series. A negative core biopsy would increase the number of cases in the nonbiopsy group, altering the result in favour of a positive impact of the biopsy procedure. Nevertheless, assuming a failure rate of less than 10% would not change our results. Secondly, SLN identification by using blue dye alone might result in a higher false negative and lower detection rate. However, this point was considered and technique of SLN identification was included in the analysis. Thirdly, preoperative hook wire localisation procedure of nonpalpable breast lesions might also cause disintegration of tumours (Fajardo, 1988). To overcome this bias, clinical palpability of the breast lesion was included in the multivariate model. The fourth point is we must emphasise that there were no data on the time interval between the preoperative biopsy procedure and the SLND available in our study. Assuming a decreased incidence of local tumour displacement at increasing intervals between core biopsy and excision, as suggested by other authors (Diaz *et al*, 1999), the impact of the time interval should bias the study in favour of an increased risk for the occurrence of occult SLN metastasis following preoperative biopsy. In addition, the timing of SLND following excision biopsy was identified as a possible confounding factor. And, finally, surgical resection itself might have an impact on tumour cell displacement and tumour cell spread. Previous studies carried out on a small number of patients revealed that manipulation during cancer surgery in human beings might result in tumour cell dissemination into the vascular circulation (Choy and McCulloch, 1996). Excisional biopsy was only performed prior to SLND in patients without preoperative diagnosis of malignancy (non-core biopsy group and patients with negative core biopsy). Assuming additional tumour cell shedding into the lymphatic system will further enhance the impact of preoperative biopsy. This point remains unclear and needs further investigation. We are indeed aware of these possible biases and the retrospective nature of the study while interpreting our data, but considering the large patient population and the power of multivariate analyses the overall impact of the above-mentioned concerns does not alter our results.

The present data clearly indicate that preoperative biopsy does not increase the risk of metastases to the SLN in patients suffering from breast cancer. There is no evidence for any tumour cell spread to the sentinel node with possible negative impact on the prognosis of breast cancer. In conclusion, preoperative breast biopsy is a safe method and should be used to achieve definitive diagnosis of malignant breast lesions.
